# The Cost of Dying Exhibition: public, professional and political reactions to a visual exhibition depicting experiences of poverty at the end of life

**DOI:** 10.1136/medhum-2024-012950

**Published:** 2024-06-24

**Authors:** Sam Quinn, Naomi Richards

**Affiliations:** 1 University of Glasgow, End of Life Studies Group Rutherford/McCowan Building, Crichton University Campus, University of Glasgow, Dumfries, UK

**Keywords:** End of life care, palliative care, Public health, sociology, exhibitions

## Abstract

Public health approaches to palliative care are internationally endorsed for their potential to improve the social determinants of dying such as energy costs, transport and housing. Enhancing public understanding of inequities in end of life experiences, which exist even in economically advanced countries, is vital if the value of public health approaches are to be endorsed and invested in. Visual exhibitions have a strong tradition of raising awareness and influencing public health discourse. The UK-based Cost of Dying exhibition (April–August 2023) presented real examples of how financial hardship and deprivation intersect with end of life experience through professional portraits, photovoice imagery taken by individuals at the end of their lives, and digital stories co-produced with bereaved relatives. Three iterations of the exhibition were displayed at public venues and a health conference. Evaluation methods comprised anonymous feedback cards (n=208), panel discussions and social media reactions. Thematic analysis was used to identify themes within the feedback. The emotional resonance of the exhibition was a key theme, with attendees expressing sadness, anger, empathy and hope. Visitors found the exhibition thought-provoking and expressed that it countered existing stereotypes about what it means to experience financial hardship at the end of life. The exhibition spurred calls for change, with some attendees questioning in what capacity they could help. Individuals with expertise in end of life care reported that the imagery validated their professional experiences. In conclusion, the Cost of Dying exhibition made visible the struggles endured by individuals confronting financial hardship and material deprivation at the end of life. Such exhibitions can challenge the traditional view of dying as a swift process taking place sequestered in institutions, revealing that it often unfolds over time and individuals may continue to live at home in the community, struggling with unmet needs and unresponsive state services.

## Introduction

 A public health approach to palliative care (Spreckley and Kellehear 2022) has been touted by a range of individuals and organisations as a means of addressing the needs of low-income communities and reducing inequalities in access to end of life care services (Hansford, Thomas, and Wyatt 2023). As defined by Prince *et al* (2022):

The public health palliative care approach is, at its core, a focus on equity, inclusivity, and diversity. The Public Health Palliative Care (PHPC) movement seeks to reclaim the natural course of dying and in the process, re-engage ‘communities’ to take on a larger role in caring for their own[…]This reclaiming requires an understanding of basic precepts that affect equity and health (Prince *et al* 2022).

Focusing on new public health approaches, this perspective advocates for addressing the broader social determinants of health—highlighting the influence of environmental, economic and social contexts on health outcomes. It underscores the importance of integrating community engagement, advancing public education on end of life matters, and mobilising civic resources to ensure equitable end of life experience across different communities (Spreckley and Kellehear 2022). The growth of public health palliative care is an international trend, with models such as the Compassionate Communities framework (Kellehear 2013) finding receptive audiences around the world (Quintiens *et al* 2022). Broadly speaking, this approach encompasses initiatives which support individuals facing serious, advanced illnesses to live out their dying in the community (and critically outside of hospitals), with a particular focus on those who are marginalised or face multiple disadvantages and are more likely to die in hospital. To do this, it is understood that the ‘death literacy’ of caregivers, service providers and the general public will need to be enhanced following many decades when lay knowledge of the dying process has been eroded (Graham-Wisener *et al *2022). Death literacy is achieved through targeted education that enhances public awareness towards death, with an emphasis on addressing disparities in perceptions and preparedness (Meier *et al *2023).

Kellehear (2019) argues that for Compassionate Communities to meet their full potential—to provide comprehensive society-wide support for advanced illness, dying and bereavement—a cultural shift is required, similar to those seen in societal discussions around disabilities (Wang *et al* 2021) and mental health (Henderson, Potts, and Robinson 2020) in the global North in the late twentieth and early twenty-first century. Such societal discussions have been characterised by a transition from stigmatisation towards normalisation, which occurred through a combination of increased public awareness, advocacy, legal reforms and a change in media portrayals (Simcock and Lee 2022).

One aspect of this ‘normalisation’ is the public dissemination of real accounts of what it is like to live with, for example, disabilities or poor mental health. Such dissemination can come via curated visual exhibitions, and there is evidence that such exhibitions can provide a platform which supports communities to confront, understand and explore mental health (Moeenrad *et al* 2022) or living with a disability (Macdonald *et al* 2022). Advocates of the Compassionate Communities movement have also argued that public exhibitions have a role to play in improving ‘death literacy’ (Leonard *et al* 2022). One example of an exhibition is the Departure Lounge project, funded by the Health Foundation (Health-Foundation 2019), which used an interactive, pop-up travelling installation to engage the public in discussions about ageing, dying and death.

While there is modest evidence exploring how public audiences have received exhibitions on the topics of dying, death and grief (Walter 2012), and on poverty (Lane 2023), there are no records of exhibitions which have explicitly explored the intersection between dying and financial hardship (Evans 2006; Moller 2012). In light of prevailing global economic challenges, the ongoing cost of living crisis in the UK, and the potential rise in compassion fatigue following the aftermath of the COVID-19 pandemic (Deng *et al *2021) there was some likelihood that the Cost of Dying exhibition would not gain traction with the general public. Compassion fatigue refers to emotional exhaustion that can occur when someone is repeatedly exposed to traumatic or distressing events (Moeller 1999).

The power of imagery of financial hardship at the end of life to cut through to the public consciousness remains underexplored. In this article, we ask what role, if any, imagery from a visual exhibition can play in enhancing public and professional knowledge about issues affecting people experiencing multiple intersecting hardships at the end of life.

## Public responses to visual exhibitions portraying dying and death

Several high-profile UK exhibitions have explored dying, death and grief in the last 20 years, leading to a broad spectrum of public responses. ‘Death: A Self-Portrait’ (2012), for example, showcased the personal collection of art collector Richard Harris, who had acquired over 2000 death and mortality-related objects. The exhibition featured artworks, artefacts, photographs and books that explored themes of anatomy, violence, commemoration and ritual (Yaqub 2012). Critically acclaimed for its diversity, richness and relevance, the exhibition garnered positive reviews and attracted over 100 000 visitors, becoming one of the most popular displays at the Wellcome Collection in London (Prigg 2012). In 2008, the ‘Life before Death’ exhibition (Schels and Lakotta 2021), also hosted at the Wellcome Collection, featured portraits taken by German photographer Walter Schels, depicting 24 people weeks before and in the hours after they had died. Schel’s partner, Beate Lakotta, also recorded interviews with the subjects in their final days. The exhibition curator James Peto explained that the exhibition elicited a strong emotional reaction in many of the 50 000 members of the public who visited (Jeffries 2008).

Most controversially, ‘Body Worlds’ (Body Worlds 2024) is a series of travelling exhibitions that display real human bodies preserved through a process known as ‘plastination’. Body Worlds opened to record crowds on 22 February 2008 at the Museum of Science and Industry in Manchester, and The O2 in London in October 2008. Ethical, legal and religious concerns have surrounded the exhibitions since the conception of the project in 1995. Criticisms include accusations of sensationalism and voyeurism (Stone 2011), while some praise them for being informative and empowering. The exhibitions are purported to have drawn more than 50 million visitors globally, making it one of the most visited in the world (Bresnahan and Austriaco 2022). Similarly ‘Real Bodies’ (Real Bodies 2024) is a display of preserved human bodies and anatomical specimens, which opened at the National Exhibition Centre in Birmingham on 8 June 2018. The exhibition aims to elicit an emotional connection and deeper understanding of life and death through immersive moving gallery displays. Concerns have been raised regarding the origin of the bodies on display in this exhibition, and whether informed consent was obtained from those who donated their bodies.

The Body Worlds (Goulding, Saren, and Lindridge 2013) and Real Bodies (Murphy 2018) exhibitions, while popular and provocative, have been criticised for their potentially dehumanising portrayal of death, focusing mainly on anatomy and science (Stone 2011). These displays, featuring skeletons and plastinated bodies, lack the context of actual dying experiences. This represents a significant gap, as visual works that intimately depict the dying process are rare (Andrec, Brookman, and Livingston 1996) (Jury and Jury 1978). Among these limited representations, the British Museum’s permanent exhibition ‘Living and Dying’ (2023) explores global cultural approaches to health and death. Its focal piece, ‘Cradle to Grave’, uses prescribed drugs woven into fabric to represent two individuals’ medical journeys. Dyer (2005) argues that the exhibitions’ contextualising text and photographs help viewers understand and reflect on the human experiences of pain and hardship.

‘The Final Project’ (2016) exhibited the late British photographer Jo Spence’s work, documenting her life and illness before her death from leukaemia. Spence, a working-class woman, used photography to confront her cancer experience, depicting the disease’s impact and employing toy skulls for modern memento mori. Her work was praised for challenging the perception of dying as solely a medical issue rather than a social and personal experience (Kenny 2015), which was helped by her inclusion of personal narratives which enhanced its emotional impact on visitors (Judah 2016). Additionally, The Bristol Museum & Art Gallery’s exhibition ‘Death: Is it your right to choose?’ explored assisted dying. It featured a replica of the Dignitas flat in Zurich, surrounded by varied opinions and personal testimonies on the subject. Graves (2016) notes its success, with over 62 000 visitors in 19 weeks and significant social media engagement, through the #isawdeath hashtag. Once again, the exhibition was commended for its use of personal narratives to foster dialogue on a key debate in end of life care.

One striking aspect of these exhibitions is the distinction between portraying death and portraying the process of dying, which is often protracted in the twenty-first century as most people die of multiple chronic conditions with death delayed through various biomedical interventions. Exhibitions that cover the topic of dying tend to be smaller and less well publicised than those related to death. This may be due to a lack of understanding or reluctance to define what ‘dying’ means. This hesitancy to define ‘dying’ might stem from the stark contrast between the certainty of what constitutes ‘dead’ and the ‘non-scheduled status passage’ which constitutes dying (Glaser and Strauss 1965). The trajectory of certain conditions, such as chronic obstructive pulmonary disease (Pinnock* et al* 2011), is difficult to predict, complicating any attempts to delineate when exactly ‘dying’ begins. Is it marked by the commencement of hospice involvement, or does it start when one is actively dying, generally considered to be within 3 weeks or less of death (Hui *et al *2014)? The lack of a cultural consensus on this matter reflects our collective unease with identifying and naming dying, which remains nebulous, unlike the finality of death itself.

This differentiation can be re-examined through Kellehear’s (2022) taxonomy of dying, which provides a more granular understanding of the dying process across different contexts. Exhibitions such as Body Worlds and Real Bodies, which display preserved bodies and medical histories, can be seen as illustrations of embodiment-led dying. In contrast, exhibitions such as The Final Project and Death: Your Right to Choose, which emphasise personal narratives and lived experiences, resonate with Kellehear’s (2022) criticism-led dying. These exhibitions use the act of dying to critique societal norms, offering a platform to explore how individual identities and social circumstances shape experiences of dying.

The Cost of Dying exhibition was designed to prioritise the individual narratives of people with lived experience of financial hardship at the end of life. Although exhibitions based on personal accounts and social identity have been generally positively received (Graves 2016), we wanted to discover how an exhibition specifically contemplating subjective experiences of poverty at the end of life would be viewed by the public, particularly at a time when the contemporary UK media was saturated by coverage of a cost of living crisis and challenging global economic conditions.

## Curation of the Cost of Dying exhibition

The purpose of this section is to detail the thought processes, discussions and practical considerations that we navigated when designing our exhibition ‘The Cost of Dying’. The audiences we were targeting included: the general public; healthcare and social care professionals (including palliative care professionals); and decision makers (both clinical and political). The first question to address was *how* to represent our visual data, the professional portraits, and our findings from the study in a way which initiated public debate and avoided didacticism and losing audience attention due to compassion fatigue.

In the study itself, we used a participatory research design to enhance participant involvement in the research process (Richards *et al* 2023). However, we knew from the outset that the curatorial decisions could not involve participants or their carers. This was because many participants had died, and those who were still alive were very sick. Bereaved carers or current carers had been involved intermittently or for discrete periods of time during the study but had made clear the limits on the time they could commit. Many carers were themselves struggling financially in addition to dealing with their grief. Aside from feasibility, there was also the question of creating a shared vision and a clear ‘take home’ message for exhibition visitors. Learning from previous research (Richards, Warren, and Gott 2012) indicated that without this shared vision, the impact would likely be lost; in seeking to serve everyone’s interests we would run the risk of serving nobody’s.

During the study, a variety of different images were created by both Margaret Mitchell and by participants. There were around 5–15 exhibitable images of each of the participants taken by the photographer. Additionally, Margaret Mitchell took 1–10 documentary images per participant. Participants also took their photovoice images; the number varied widely by the participant, with some individuals taking no photographs while others took up to 150 photovoice images. Finally, three digital stories were created by bereaved carers.

The research team held several video calls about how these different types of images, as well as the digital stories, would be presented alongside one another within the same venue space. How images are juxtaposed changes their meaning (Metcalfe 2015), and there were particular sensitivities around juxtaposing images taken by participants to represent their subjective ‘insider’ perspective and the professional portraits taken with an artistic ‘outsider’ sensibility (Richards* et al* 2023). There were also deliberations about how much sociological context to give on the general topic of the structural determinants of dying, biographical and clinical details about each participant, and how much to let the images do the heavy lifting and allow for audience interpretation; in other words, how much didacticism to introduce.

Three different venues were selected to appeal to our different target audiences. The venues, audiences and sources of feedback we solicited at each venue are presented in table 1:

**Table 1:** Exhibition details and types of data collected

Exhibition location	Length	Target audience	Design	Feedback collected
Palliative Care Congress, Edinburgh	16–17 March 2023(2 days)	Health and social care professionals; palliative care professionals; clinical researchers	Thematic	Written feedback cards from palliative care professionals and clinicians (n=16) where a smaller version of the exhibition was previewed.
Advanced Research Centre, University of Glasgow	25 April to 5 May 2023(10 days)	The general public; politicians and decision-makers; academic researchers; university students	Individual stories	Written feedback from attendees (n=118) over the 10-day exhibition. The audience included the participant’s family, friends and carers, palliative care professionals, social sector representatives, academics, students, and the general public.A cross-sector panel discussion was also held during the exhibition, where experts discussed their responses to the images (audience n=49, panel n=4).
The Mitchell Library, Glasgow	20 June to 19 August 2023(2 months)	The general public; politicians and decision-makers	Individual stories	Written feedback from the general public (n=74).A cross-sector panel discussion was also held during the exhibition at this venue, where experts discussed their responses to the images (audience n=35, panel n=4).

Through a series of video calls, the whole research team, including the photographer, met to discuss the selection of professional images to show at the different exhibitions. Margaret Mitchell’s image selection was respected in most cases based on her professional view about which were the visually strongest images. There were a couple of images where there was a difference of opinion in terms of the researcher’s view of what were better or lesser known visual tropes of dying in end of life policy and artistic discourse. This involved tense discussions with each team member’s rationale being articulated, considered, and a compromise eventually forged.

A third ‘voice’ was brought into the mix by hiring an exhibition producer. Oona Dooley was brought onto the team to help with designing the text panels so that they were of professional quality and to ensure that language and typeface were accessible. She also had considerable experience working with visual artists, accommodating their vision, and balancing a range of media into a cohesive exhibit. This strategy is to be recommended for other research teams designing exhibitions, as it is important to draw on those with dedicated expertise and knowhow to produce something which is ‘read’ by audiences, whether consciously or not, as a ‘professional’ exhibition and therefore deserving of their considered attention and dedicated time.

An early key decision to be made was whether or not to present our participants’ stories individually or to present findings thematically. While on the one hand, we recognised that the first presentational style would better reflect the subjectivities and nuances of each participant’s story, on the other, we were concerned that the larger structural cross-cutting issues arising across the sample might be less perceptible to an audience. Our traditional academic outputs (journal articles, conference presentations) presented the study findings thematically, seeking to give a general account rather than to evoke people’s individual stories in the round (Richards *et al* 2024). However, for the exhibition, our main aim was to evoke emotional identification from visitors using the specificities of the participants’ lives. In the end, we designed two exhibitions—one thematic (with some hint of the personal stories involved) for our mainly clinical audience, and one entirely individually focused exhibition for our general public audience (including politicians).

**Figure 1:**
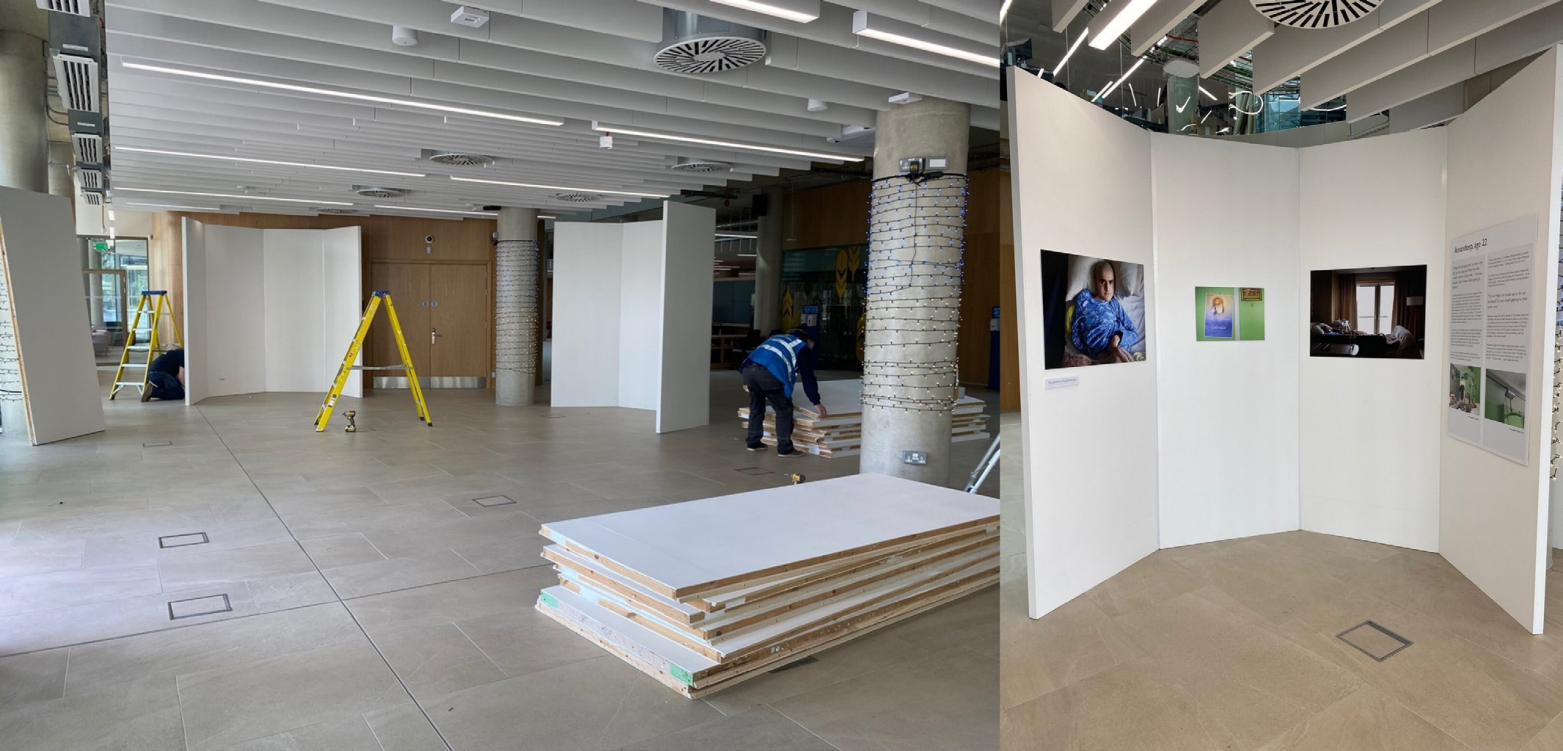
Half-hexagon ‘nooks’ under construction using large white display boards to create a temporary, more enclosed and thus more private space to view images in the Advanced Research Centre. Copyright Dying in the Margins, 2023 all rights reserved.

The individually focused public exhibition was organised in a series of eight half-hexagon four-sided ‘nooks’ using large, high-quality display boards (bought in at considerable cost by the university). This orientation was originally proposed by Margaret Mitchell at an early recce of the space. An example of the ‘nooks’ and how they functioned to create more private, intimate space within a vast open area of a modern university building can be seen in figure 1. We felt the half hexagon ‘nook’ formation would also restrict the number of visitors who could be immersed in each individual’s story at any one time, again, creating intimacy and enhancing imaginative identification with the stories (figure 1).

**Figure 2:**
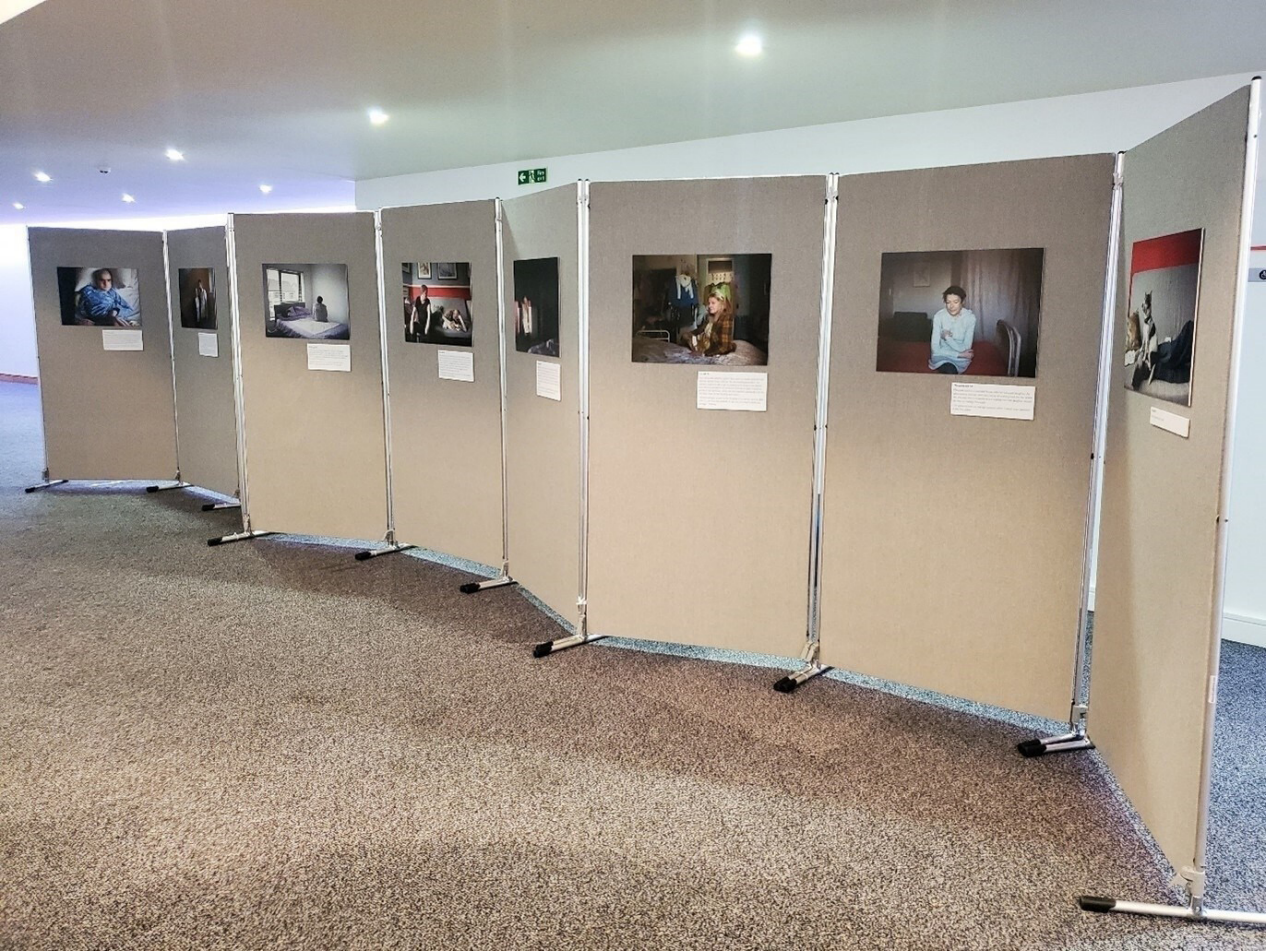
Portrait images exhibited at the conference. Copyright Dying in the Margins, 2023 all rights reserved. Pictured portraits: Copyright Margaret Mitchell, 2023 all rights reserved.

The thematically structured exhibition for a predominantly clinical audience of palliative care researchers and practitioners was more circumscribed in terms of the scope available to create a professional aesthetic. The exhibition venue was a traditional corporate conference space and we had to use conference poster boards (supplied by the venue) to display the images. Portrait images with small text captions were displayed on one side (figure 2) while the thematic text panels, including additional documentary, photovoice images and participant quotes, were displayed on the other side (arranged by our exhibition producer). In sum, while we needed to target a clinical audience for the images and study findings, the set and setting were more corporate and had a less traditional art exhibition aesthetic than the individually structured exhibition, and emotional identification with the images and stories was potentially reduced (see figure 2).

**Figure 3:**
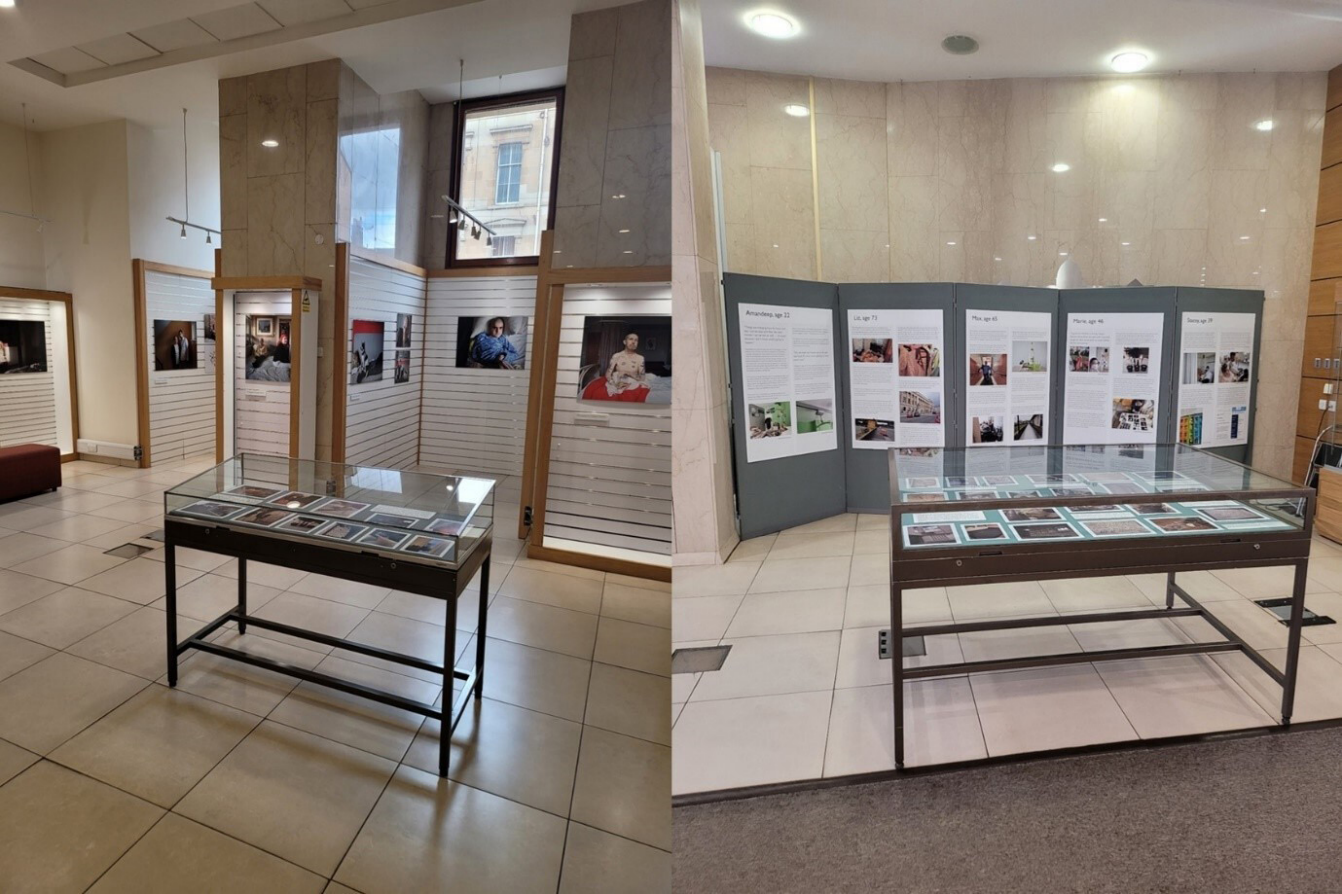
The library display featuring glass cabinets with photovoice images. Copyright Dying in the Margins, 2023 all rights reserved.

In the Mitchell Library exhibition space, we needed to reuse the already printed materials and adapt the format for the space. While this exhibition remained individually structured, the ability to come up with a bespoke design for a very open space which acted as a thoroughfare in its location near a main door, meant that the intimate feel of the earlier exhibition was lost. The venue was chosen to target a specifically public audience, and as one of the largest libraries in Europe with an estimated 400 000 visitors per year, we anticipated significant footfall over the exhibition’s 2-month-long duration. The main addition made to this version of the exhibition was the introduction of more participant photovoice images, arranged in glass display cases by the exhibition producer (see figure 3).

## Evaluation of the exhibition

In setting out to evaluate the Cost of Dying exhibition, we were guided by an overarching research question: What are the responses of different audiences to a photographic exhibition depicting experiences of financial hardship and deprivation at the end of life?

Aesthetic experiences are hugely subjective and exhibition visitors’ experiences are shaped not just by visual elements but also by a broad spectrum of experiential factors (Vissers and Wagemans 2023). The ‘How To: Evaluate an Exhibition’ framework from Cambridge Museums (Noble 2018) provides a foundational approach for assessing the impact of visual exhibitions accounting for these experiential factors, highlighting methods such as surveys, interviews, observations and ethnographic notes. Reflecting this methodological diversity, various evaluation studies have adopted different approaches. Merrett and Eveleigh (1985), for example, administered quantitative surveys to visitors before and after they viewed the ‘Why Worry?’ exhibition to evaluate the exhibition’s impact on anxiety awareness. Ballarini, Mougenot and Prokupek (2019) employed semistructured spot interviews to evaluate the ‘DeTermination’exhibition, and (Lakhanpaul *et al* 2021) took a mixed-methods approach to evaluating the ‘Birthing a Better Future’ exhibition. Given the diverse methodologies highlighted in the literature, it is clear there is no ‘gold standard’ or universally accepted best practice for exhibition evaluation. Instead, the choice of method often depends on what is feasible and most appropriate for the specific context and objectives of the exhibition being assessed.

The evaluation of the Cost of Dying exhibition consisted of an anonymous feedback box, two cross-sector panel discussions and comments posted on social media. Anonymous feedback forms are a well-established method in the evaluation of art gallery and museum exhibitions (Felten and Kahn 2021) as they provide an efficient way of collecting a large amount of qualitative feedback (Noble 2018). However, it is important to acknowledge that this method produces a self-selective sample, and visitors may be more likely to post comments if they feel strongly about the issues covered (Elston 2021).

Second, two cross-sector panel discussions were hosted: one at the University exhibition (n=49 attendees), and the second at the community exhibition (n=35 attendees). Four panel members participated at each event and discussed their responses to the images and how services could be improved for people experiencing financial hardship at the end of life. Panellists included bereaved family/carers, palliative care practitioners, social sector professionals and policy experts. Both events were open to a public audience and all attendees were made aware that the event was being audio recorded for later analysis.

Finally, we scanned online media coverage (BBC News website) and social media (Facebook, Instagram and Twitter) for responses to the coverage of the exhibition. These comments were tabulated in Microsoft Excel and included in the final analysis. Data were collected and analysed from the sources presented in table 1.

Over 200 pieces of feedback were received across the three exhibitions. In some cases, it was possible to recognise the job designation of the commenter. Additionally, some individuals self-disclosed their occupation or mentioned that they were a family member of a participant. The data from the exhibition evaluation were analysed using thematic analysis as per Clarke and Braun’s (2013) methodology, a process focused on identifying, analysing and interpreting patterns within qualitative data. Initially, the researcher, SQ, familiarised themself with the feedback. They then generated initial codes by methodically identifying notable aspects of the data. These codes were organised into potential themes, which were reviewed by NR and refined to ensure they accurately represented the exhibition evaluation feedback. Quotations are included to exemplify the identified themes.

### Patient and public involvement statement

Patients and the public were involved in the conduct and dissemination of our research, contributing valuable insights through the photovoice method and public feedback on the exhibition, which shaped the ongoing curation and presentation of the Cost of Dying exhibition.

## Reception of the images

The analysis produced the following four key themes: the emotional impact of the exhibition; challenging stereotypes and education; call to action; and desire for change. Less prevalent themes were how the exhibition affirmed the experiences of professionals and family carers and practical considerations.

### Emotional impact of the exhibition

**Figure 4:**
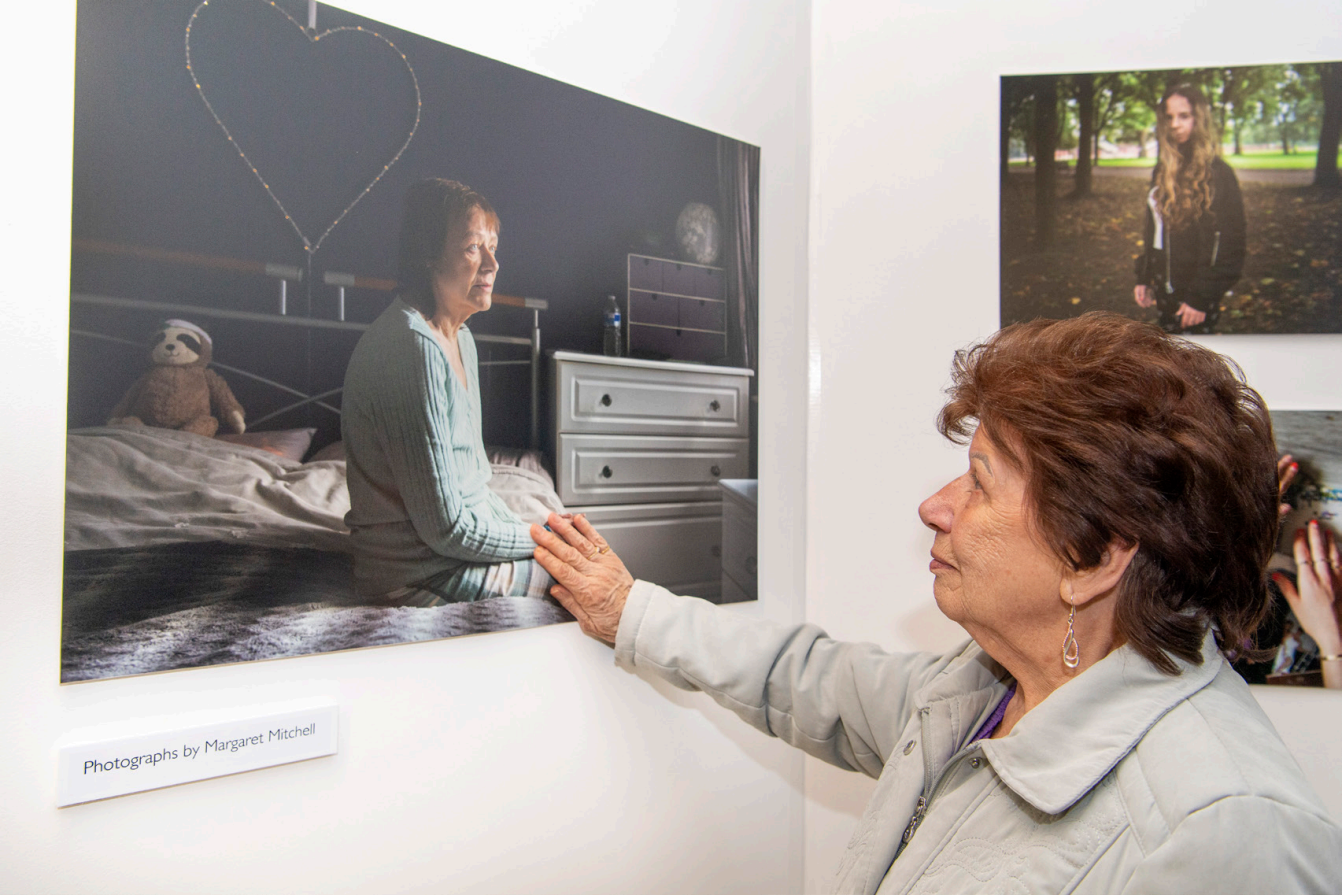
A participant’s mother views a picture of her daughter. Permission to use the image was sought and provided by the University of Glasgow Photography Unit and the individual pictured. Pictured portrait: Margaret Mitchell, 2022 all rights reserved.

The emotional impact of the exhibition was very prominent in the feedback received, although the nature of the emotion differed widely. Those who identified themselves as family members reported a strong sense of pride in their relative’s involvement (see figure 4), a feeling that their relative had been selfless in sharing their experiences and that the images constituted part of their legacy:

That sense of pride that they, at a time when they were facing a huge amount of other challenges, death being one of them, because they knew that it was coming, that they would consider a legacy, of leaving a legacy and that legacy being that there are issues that need to be addressed. That [my niece], you know, wasn’t considering what people would think about her when her story was being told, but what her story would mean for us and helping to prevent others going through the same thing. (Family member at panel discussion)

The panel member’s mention of her niece, who ‘wasn't considering what people would think about her’, alludes to the stigma surrounding financial hardship. Throughout the study, we grappled extensively with questions around which images to make public due to concerns about contributing to stereotypes of poverty and of the potentially shaming effect for those we have worked with. In a cultural context where even the term ‘poverty’ has become highly politicised and certainly disowned as a social identity (Hudson 2015), showing images of the lives of people affected by poverty and deprivation is also a highly politicised undertaking.

For one, there is wide scope for misinterpretation by audiences, including those reading this article, and a potential closing down rather than an opening up of viewers’ empathy. Our confidence in releasing these images comes down to three guiding ethical principles. The first is that our participants wanted their stories to be told to improve care for people dying in the future. While levels of engagement and commitment varied, all were aware from the very first conversation that the images were destined for a public audience with the intention to capture the imagination of decision-makers and effect real-world change. The second principle is that we strive to be balanced in what we make public in order not to be reductive about participants’ lives and reduce them to single issues. The final principle which guides us is the idea that by not showing some of the more challenging or stereotypical images we are potentially colluding with ideological forces which deny the existence of poverty or blame individuals for their circumstances, as well as potentially undermining the autonomy of our participants who took the images with the intention of showing them.

Some members of the public also found the images profoundly emotional, with sadness being prominently expressed:

i am so sad that the country that i love has such inequality - from birth to death. (advanced research centre feedback)

However, in some cases, initial feelings of sadness gave way to anger that, in a comparatively wealthy country, people were dying in poor financial and material circumstances:

I hesitated to visit this exhibition as I felt it might just be too depressing. But it was not. The dignity and beauty in Margaret Mitchell’s portraits mitigated against the sadness. I am, however, leaving the exhibition angry that in a wealthy country such as ours, people are not being given the opportunity to die with dignity in their own homes. (Advanced Research Centre feedback)

There was also a strong undercurrent of appreciation and empathy for the individuals who shared their experiences of financial hardship at the end of life:

Thank you to the brave people who allowed themselves to be included, sharing their experiences. (Library feedback)

There were also mentions of hope and optimism that things will improve:

A very powerful and moving exhibition. Just hope and pray that more will be done for these courageous people as a result. (Library feedback)

We had previously questioned if the intense media focus on the cost-of-living crisis and the negative economic outlook might lead to compassion fatigue, potentially reducing public interest in the exhibition. However, our data analysis and the feedback received suggest otherwise. Many attendees reported that their visit was prompted by the BBC’s coverage of the exhibition (Keenan-Bryce 2023). This indicates that, despite the challenging economic climate, the media exposure helped maintain public engagement rather than contributing to compassion fatigue. It’s important to note, however, that the feedback mainly came from self-selected individuals already interested in the exhibition.

Saw this on the news, very moving. A time when money worries shouldn't come first but do. Thank you to all those that took part. (Social Media feedback)

A bit morbid and macabre but it’s a part of life I suppose. (Social Media feedback)

To gain a broader perspective, we also included social media comments from Facebook, Instagram and Twitter in our thematic analysis. Although a small minority found the subject matter too upsetting or morbid, the majority of comments on social media underscored the exhibition’s emotional impact, often highlighting how it deeply resonated with visitors.

### Challenging stereotypes and education

It has been argued that only by normalising discussion of dying, death and grief in our everyday lives can death literacy be improved and the foundations for a public health approach to palliative care be laid (Kellehear 2020). This includes the need to diversify accounts of how different intersecting characteristics such as gender, race, ethnicity, sexuality, disability and socioeconomic status can shape end of life experiences (Grindrod 2020). Members of the public as well as palliative care practitioners noted that the exhibition shed light on an unseen section of society, challenged prior assumptions of what it means to be experiencing poverty while dying, and made them reflect on their own lives and privileges:

The imagery, I felt for me, really captured the kind of personal experiences of people going through it…. [It] really brought home, this is what it is for individuals, this is what it is for people going through it, taking it out from behind the door and highlighting that, you know, it’s not always quietly, comfortably, happily. (Practitioner at panel discussion)

Really informative about areas I've not had much experience of due to my extremely lucky privilege. (Conference feedback)

Palliative care professionals tended to view the exhibition as a ‘creative way to disseminate research’, and a helpful way to ‘triangulate [understanding] with the numerical and population data’—in other words as a complement to more positivistic methods used in clinical research.

Approaches to influencing public behaviours and attitudes vary significantly in their approach. Governmental and policy-making bodies often employ top-down strategies (Buse, Mays, and Walt 2005), using policy-driven methods to guide public behaviour. In contrast, educational institutions and social campaigns tend to focus on changing attitudes through educational means and empathic appeals. There is significant evidence to suggest that emotionally oriented education can trigger empathy and increase pro-social behaviour (Dirkx 2001; Potash* et al* 2013). As such, we argue that it was the emotional impact of the Cost of Dying exhibition, particularly the elicitation of empathy among attendees, which was the key mechanism for educating professionals, policy-makers and the public. The connection between emotional identification and the educational import of the exhibition was prevalent in the feedback:

[The exhibition] showed the patient’s humanity in a way the viewer could identify with easily whilst bringing home the reality of the situation. Very respectful of the participants and thought-provoking for the viewer. I suspect that many doctors are unaware of the poverty their patients face and that knowledge from the general public is even more limited. (Advanced Research Centre feedback)

Members of the public identified that they were previously unaware of the challenges faced by people experiencing poverty at the end of life, particularly issues around housing:

It made me reflect on the impact of housing and apartment living on end of life care. Really interesting to learn about the role hospice is playing in caring for people who are financially struggling. (Advanced Research Centre feedback)

There is good quality evidence from movements such as the animal rights movement (Green *et al *2019) that visual exhibitions can elicit emotional engagement which can provide a launch pad for education and inspiring action. However, there is the possibility that attendees may feel engaged and motivated, but then feel a sense of powerlessness or paralysis from being unsure of how to help or bring about change:

Wish there was a way for viewers to help… Would love to know if there are any volunteering opportunities especially some organisations that help in dealing with loneliness and isolation. (Library feedback)

It is therefore important to consider what is being asked of attendees. While our curatorial intent was to create a non-directive exhibition which did not lead the audience in an overtly didactic way, but rather left room for viewer interpretation, this had the effect of leaving some people feeling unmoored at the end of the exhibition. While we could have elected to signpost volunteering opportunities or explicitly advocate for specific policy changes and directed visitors to register their support for those changes, this would have markedly changed the tone of the exhibition.

### Call to action and desire for change

The exhibition was seen as a call to action, with some attendees expressing hope that it would lead to improvements in end of life care and policy changes. The feedback did not tend to identify specific policy recommendations but rather respondents expressed a desire for the exhibition’s message to reach a broader audience and to be cascaded far and wide. The comments suggest that the exhibition had the potential to influence policy and practice by raising awareness and promoting change in the way society addresses end of life care and poverty issues:

OMG. This is desperate stuff! I hope this has a much wider audience - academics, media, political classes. (Library feedback)

Attendees’ recommended improvements in public services, joint working between healthcare and social care, and the importance of an adequately funded social care system:

Public services need to improve to support people facing the end of life. Transport, social security, and housing, are all not good enough. (Advanced Research Centre feedback)

Professionals and carers at the panel discussions highlighted several specific areas for policy improvement. These included increasing resource allocation to primary care in more deprived areas to address health disparities and enhancing access to quality care. Additionally, there was a call to expand the community link worker programme in Scotland (Mercer *et al *2019) and increase the number of financial advisors in General Practitioners (GP) surgeries. Another key recommendation emphasised the need to optimise welfare assessment forms such as those specifically designed to streamline and amplify financial entitlements for individuals with terminal illness (Social Security Scotland 2023).

There was some discrepancy over whose responsibility it is to bring about positive change for people facing financial hardship at the end of life. While some of the feedback received critiqued the National Health Service (NHS), housing and social care sector, a similar level of critique was directed at successive governments for generally weakening the welfare state:

I believe the introduction panel should directly call out both the UK and Scottish governments rather than the NHS, housing and third sector. The welfare state has been systematically dismantled over decades. (Advanced Research Centre feedback)

Reflecting on the exhibition, a framework proposed by Khatibi *et al* (2021) for creating social change through community engagement can be applied. This model delineates a progression from awareness-raising, which is about informing the public on a specific issue, to public engagement, where this initial awareness evolves into active involvement and discourse. In the evaluation of the Cost of Dying exhibition, this pathway can be observed. Feedback suggests that some members of the public had not previously considered the complexities and challenges faced by people experiencing financial hardship at the end of life. This awareness then transitioned into a deeper level of engagement, as evidenced by queries regarding potential improvements for people in these circumstances. While some attendees suggested more explicit calls to action or a more politicised tone, other feedback supports the original, more politically nuanced curatorial approach, in which we attempted to allow the images and research findings to speak for themselves.

### Affirming personal experience

A minor but important theme was affirming personal and professional experiences. This applied to many practitioners and people with expertise in end of life care who reported seeing their professional experiences depicted in the imagery:

I've nursed so many people in so many different circumstances toward the end of their life, that at times it has been my compassion and love that’s been the main staple, as they have been let down by doctors, local authorities and the agencies meant to provide them with care until the end. (Advanced Research Centre feedback)

Many are stories I have heard or been involved in, but in a similarly deprived area of England. (Conference feedback)

Additionally, some members of the public shared personal connections to the themes of the exhibition, including experiences of loss and life-limiting illness:

My father died 37 years ago from cancer - we had no help. It’s shocking that, in financial and social ways, things have moved so little. (Advanced Research Centre feedback)

### Practical considerations

Most of the critiques received were not issues with the subject matter itself but were practical considerations about the way the exhibition was set up or organised. Where possible, we sought to address criticism in future exhibitions. One key request was for the 10-day exhibition to be on for longer. Fortunately, we were able to address this by hosting the images in the community venue for 2 months. In another instance, a visitor to the Advanced Research Centre commented:

Margaret Mitchell’s photos were very beautiful, but I would like to see more of the photovoice as well. (Advanced Research Centre feedback)

Responding to this, we made a concerted effort to include more photovoice at the Mitchell Library exhibition, presenting photovoice prints in two large glass-top display tables (see figure 3). Other visitors critiqued some environmental issues within the locations of the two larger exhibitions.

There is an emptiness surrounding the exhibition. It is too spread out. (Library feedback)

Exhibition area could have been placed in a quieter location, trolleys being moved, people laughing in other rooms while reading people’s stories of end of life perhaps inappropriate. (Advanced Research Centre feedback)

While one attendee suggested it may be inappropriate to hold an exhibition about dying in a busy, public venue where other events are being held, there is a case to be made for having done so. First, holding the exhibition in such a location maximised footfall, allowing more people to engage with participants’ stories. Second, by incorporating an exhibition about dying into a busy work environment, we perhaps went some way towards normalising the topic and rejecting the need to speak in hushed tones or in sequestered spaces.

While the choice of venue for the exhibition received some minor critique, the overall layout and the unique 'nook' arrangement of the space garnered praise. A panel discussion participant highlighted this aspect:

The way that each one is a shape like a curve. You enter into that person’s space, but the space is … respectful and clean and a light colour, so there’s no distraction from the personhood that’s being expressed in each section. So, each person is in their own space with their own privacy. (Academic palliative care clinician at panel discussion)

Overall, this feedback highlights the importance of carefully considering in advance (and being able to justify after the fact), the way that an exhibition is arranged and curated in the space, although each venue will bring both unique challenges and benefits, and adaptations and compromises will need to be made along the way.

## Conclusion

Enhancing public understanding and awareness of end of life experiences across socioeconomic divides is essential for developing effective public health palliative care strategies. The Cost of Dying exhibition demonstrated the compelling power of visual story-telling to highlight the intersecting challenges of poverty, material deprivation and dying. This approach fosters empathy and reflection and challenges societal norms around dying, urging a shift towards more inclusive and culturally sensitive care.

Our findings underscore the profound impact of combining professional imagery and personal narratives in raising awareness and sparking dialogue on previously underexplored aspects of dying. Despite initial concerns about compassion fatigue or perpetuating stereotypes of poverty, the feedback received reveals a strong interest in such intimate portrayals of end of life experiences. This highlights the necessity of incorporating visual exhibitions as a core component of public health initiatives.

To advance a public health approach to palliative care, it is crucial to broaden our narratives to encompass a diverse range of life experiences, particularly from those who are structurally disadvantaged. By doing so, we not only enrich our collective understanding of dying but also promote a broader societal engagement with end of life issues, fostering a shared responsibility for compassionate care.

## Data Availability

Data are available upon reasonable request.
