# Correction: *The cost of dying exhibition: public, professional and political reactions to a visual exhibition depicting experiences of poverty at the end of life*

**DOI:** 10.1136/medhum-2024-012950corr1

**Published:** 2025-10-22

Quinn S, Richards N. The Cost of Dying Exhibition: public, professional and political reactions to a visual exhibition depicting experiences of poverty at the end of life. *Med Humanit* 2025;51:207-217. doi: 10.1136/medhum-2024-012950

This article was corrected after publication with the agreement of the authors. The captions of figures 1–3 have been amended to include full copyright details. Figure 4 was removed from the article for privacy reasons.

